# Secondary Acute Myeloid Leukemia in Treatment-Naïve Primary Testicular Diffuse Large B-Cell Lymphoma

**DOI:** 10.1155/crh/9885915

**Published:** 2025-10-28

**Authors:** Hany Haqimi Wan Hanafi, Nurul Miftah Mohd Sabri, Rosmaniza Muhamat Yusoff, Nurul Asyikin Nizam Akbar, Noor Haslina Mohd Noor, Faezahtul Arbaeyah Hussain, Azlan Husin

**Affiliations:** ^1^Clinical Hematology Unit, Department of Internal Medicine, School of Medical Sciences, Universiti Sains Malaysia, Kubang Kerian, Kelantan, Malaysia; ^2^Hospital Pakar Universiti Sains Malaysia, Kubang Kerian, Kelantan, Malaysia; ^3^Department of Hematology, School of Medical Sciences, Universiti Sains Malaysia, Kubang Kerian, Kelantan, Malaysia; ^4^Department of Pathology, Queen Elizabeth Hospital, Kota Kinabalu, Sabah, Malaysia; ^5^Department of Pathology, School of Medical Sciences, Universiti Sains Malaysia, Kubang Kerian, Kelantan, Malaysia

**Keywords:** clonal evolution, diffuse large B-cell lymphoma, secondary acute myeloid leukemia, testicular lymphoma

## Abstract

Secondary acute myeloid leukemia (sAML) typically arises from a prior myeloid malignancy or as a complication of cytotoxic therapy for other cancers. Rarely, it may develop without antecedent treatment, particularly in lymphoid malignancies. We report an unusual case of sAML in a treatment-naïve patient previously diagnosed with primary testicular diffuse large B-cell lymphoma (DLBCL). A 58-year-old male initially presented with Stage 1E primary testicular DLBCL and declined recommended treatment. Five years later, he developed symptoms and laboratory features of acute myeloid leukemia (AML), confirmed as monocytic subtype (M5) via immunophenotyping. Despite planned hypomethylating agent-based therapy, he succumbed during bridging treatment. This case highlights the diagnostic importance of immunophenotyping and an uncommon clinical trajectory from untreated lymphoid malignancy to sAML.

## 1. Introduction

Secondary acute myeloid leukemia (sAML) is a distinct subtype of acute myeloid leukemia (AML) that arises either from an antecedent hematologic disorder such as myelodysplastic syndromes, myeloproliferative neoplasms or aplastic anemia, or as a consequence of prior exposure to cytotoxic chemotherapy or radiation therapy, termed as therapy-related AML [1]. Primary testicular lymphoma, most commonly diffuse large B-cell lymphoma (DLBCL), is an aggressive yet rare extranodal lymphoma constituting only 1%-2% of all non-Hodgkin lymphomas [2]. Transformation or evolution of DLBCL into a leukemic phase is exceedingly rare, especially in treatment-naïve patients. We present a unique case of sAML arising 5 years after a diagnosis of untreated primary testicular DLBCL.

## 2. Case Presentation

**Figure 1:**
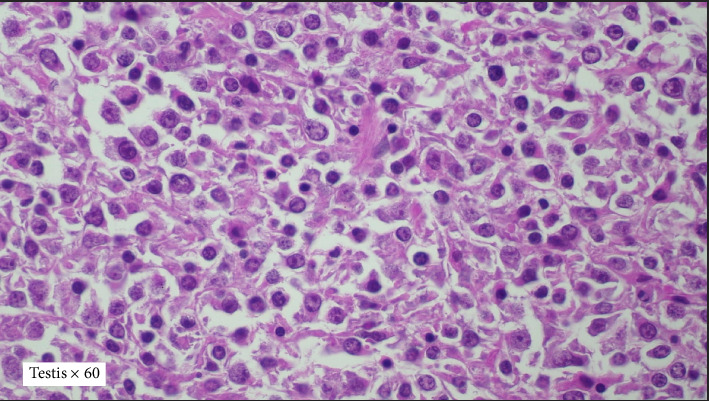
Extensive infiltration of testicular tissue by sheets of atypical lymphoid cells. The malignant cells are moderate to large with vesicular nuclei, prominent nucleoli, and scanty cytoplasm (H&E stain, magnification × 60).

**Figure 2:**
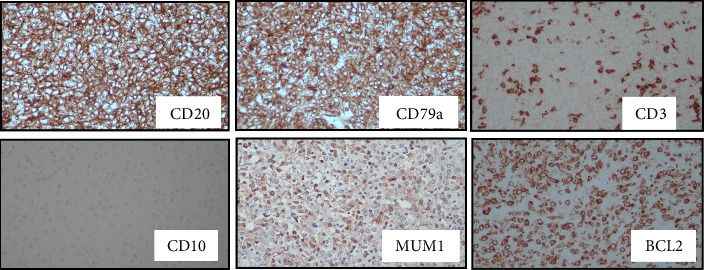
IHC of the testicular tissue showed positivity toward CD20 and CD79a, while absence of the CD3 marker on small lymphocytes. The second line IHC showed negativity toward CD10 and positivity toward MUM1 and BCL-2, hence consistent with nongerminal center subtype of DLBCL.

A 58-year-old male initially presented with painless right testicular swelling. Histopathology analysis of the right orchidectomy specimen revealed diffuse infiltration of the testicular tissue by sheets of atypical lymphoid cells (Figure 1). Immunohistochemistry (IHC) confirmed the diagnosis of DLBCL, nongerminal center subtype, with positivity for LCA, CD20, CD79a, MUM1, and BCL2, and negativity for CD3 and CD10 (Figure 2). Contrast-enhanced CT showed no significant nodal or visceral involvement. Bone marrow biopsy was negative for lymphoma infiltration. A diagnosis of Stage 1E primary testicular DLBCL was made. Despite being fit for R-CHOP chemotherapy and intrathecal chemotherapy prophylaxis, the patient declined treatment and was lost to follow-up.

**Figure 3:**
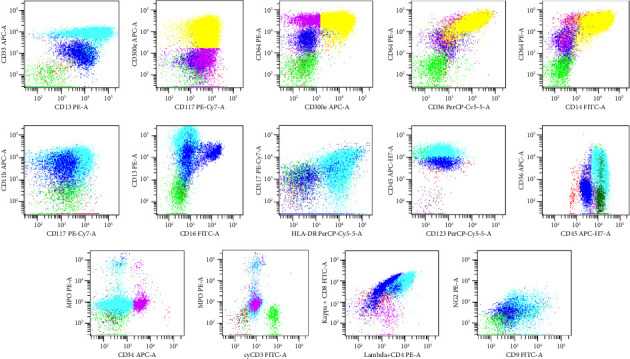
The immunophenotyping showed a predominant population with bright CD45 expression and high side scatter. This population of cells expressing monocytic and myeloid markers includes CD13, CD33, CD117, CD11b, CD16, HLA-DR, CD300e, CD36, CD14, CD56, and CD123. The cells were negative for CD34, MPO, NG2, and lymphoid markers (CD3, CD4, and CD8), with no evidence of light chain restriction.

Five years later, he re-presented with a 3-week history of lethargy. Physical examination showed no lymphadenopathy, hepatosplenomegaly, or testicular abnormality. Laboratory tests revealed severe anemia (Hb 6.7 g/dL), leukocytosis (WBC 58.7 × 10^9^/L) with 43% circulating blasts, and normal platelets. Bone marrow aspirate demonstrated 60% blasts, while immunophenotyping by flow cytometry revealed a predominant population with bright CD45 expression and high side scatter, positive for monocytic and myeloid markers including CD13, CD33, CD117, CD11b, CD16, HLA-DR, CD300e, CD36, CD64, CD14, CD56, and CD123. The cells were negative for CD34, MPO, NG2, and lymphoid markers, with no evidence of light chain restriction (Figure 3). Cytogenetic analysis revealed a normal male karyotype. Unfortunately, molecular analysis such as next-generation sequencing (NGS) of FISH was not feasible due to financial and logistical constraints, limiting the evaluation of shared genetic alterations between the initial lymphoma and subsequent AML. Imaging showed mildly enlarged lymph nodes that were inaccessible for biopsy. The final diagnosis was sAML with monocytic differentiation (M5), arising in a patient who, at this time of diagnosis, was burdened by multiple comorbidities including Type 2 diabetes, hypertension, atrial fibrillation, and Stage 4 chronic kidney disease.

The patient was planned for azacitidine and venetoclax but initiated on low-dose cytarabine as bridging therapy due to frailty. Unfortunately, he deteriorated rapidly and passed away before definitive treatment could begin.

## 3. Discussion

The development of sAML in patients with lymphoid malignancies is uncommon, particularly in the absence of prior cytotoxic therapy. Most transformations involve lymphoid malignancies usually with advanced stages of disease, under cytotoxic pressure [3]. Our case suggests a possible intrinsic genomic instability or clonal evolution leading to myeloid transformation in the absence of therapy.

The differentiation between blast-phase lymphoma and AML is critical, particularly when immunophenotypic profiles overlap. Flow cytometry remains a cornerstone in delineating lineage and guiding diagnosis. The immunophenotype demonstrated expression of monocytic-associated markers including CD14, CD36, CD11b, CD300e, and CD123, with absence of lymphoid markers and myeloperoxidase, supporting a diagnosis of AML with monocytic differentiation (M5 subtype).

In cases of AML in older or frail patients, hypomethylating agents such as azacitidine combined with BCL-2 inhibitors such as venetoclax have emerged as an effective and tolerable frontline option [4]. Although this patient was planned for such therapy, he unfortunately succumbed during bridging treatment. This highlights the urgent need for early identification and prompt initiation of therapy in high-risk sAML cases.

A few cases of simultaneous diagnoses of both lymphoid malignancies and AML have been reported. However, the development of sAML following untreated lymphoid disease, particularly DLBCL, remains extremely rare. Stern et al. described three cases of chronic lymphocytic leukemia (CLL) managed with watchful waiting that eventually progressed to AML [5]. Similarly, Ito et al. reported a small number of untreated CLL patients who developed sAML [6]. These observations support the hypothesis that in certain individuals, clonal hematopoiesis or underlying genomic instability may predispose to lineage switching or dual hematologic malignancies [7]. Although rare, the literature supports instances of AML developing after lymphoid malignancies, particularly in patients with CLL or relapsed lymphomas under treatment pressure. However, transformation from untreated testicular DLBCL remains exceptionally uncommon [6, 7]. A formal systemic review of similar cases was beyond the scope of this report; however, available case reports suggest variable latency, frequent monocytic differentiation, and poor prognosis in similar transformations.

Emerging evidence suggests that specific genetic alterations in DLBCL may predispose patients to clonal evolution and subsequent development of sAML, even in the absence of cytotoxic therapy. Notably, DLBCL often harbors mutations in genes involved in immune evasion and NF-κB pathway activation, such as MYD88 and CD79B, which contribute to sustained B-cell receptor signaling and oncogenic progression [8]. While a direct link between these mutations and secondary leukemogenesis has not been fully established, their role in promoting genomic instability may artily explain the rare transformation observed in this case.

## 4. Conclusion

This case illustrates a rare progression of untreated primary testicular DLBCL to sAML. It emphasizes the value of comprehensive diagnostic workup, especially flow cytometry, in distinguishing between lymphoid and myeloid processes. Greater awareness is needed for atypical transformations in lymphoma patients, even without cytotoxic exposure. This case highlights the importance of long-term follow-up in untreated lymphoma patients and raises questions about surveillance strategies and the biological link between lymphoid and myeloid malignancies. Clinicians should remain vigilant for atypical disease evolution even in the absence of initial therapy.
